# Functional and Symptomatic Individuality in the Response to Levothyroxine Treatment

**DOI:** 10.3389/fendo.2019.00664

**Published:** 2019-09-26

**Authors:** Rudolf Hoermann, John E. M. Midgley, Rolf Larisch, Johannes W. Dietrich

**Affiliations:** ^1^Department for Nuclear Medicine, Klinikum Lüdenscheid, Lüdenscheid, Germany; ^2^North Lakes Clinical, Ilkley, United Kingdom; ^3^Medical Department I, Endocrinology and Diabetology, Bergmannsheil University Hospitals, Ruhr University of Bochum, Bochum, Germany; ^4^Ruhr Center for Rare Diseases (CeSER), Ruhr University of Bochum and Witten/Herdecke University, Bochum, Germany

**Keywords:** intra-class correlation, response heterogeneity, LT4 treatment, thyroid carcinoma, thyroid homeostasis, setpoint, ergodicity

## Abstract

**Background:** For significant numbers of patients dissatisfied on standard levothyroxine (LT4) treatment for hypothyroidism, patient-specific responses to T4 could play a significant role.

**Aim:** To assess response heterogeneity to LT4 treatment, identifying confounders and hidden clusters within a patient panel, we performed a secondary analysis using data from a prospective cross-sectional and retrospective longitudinal study.

**Methods:** Multivariate and multivariable linear models adjusted for covariates (gender, age, and BMI) were stratified by disease-specific treatment indication. During follow-up, pooled observations were compared from the same patient presenting either with or without self-reported symptoms. Statistical analysis was extended to multilevel models to derive intra-class correlation coefficients and reliability measures during follow-up.

**Results:** Equilibria between TSH, FT4, and FT3 serum concentrations in 342 patients were examined by treatment indication (benign goiter, autoimmune thyroiditis, thyroid carcinoma), consequently displaying complex interactive response patterns. Seventy-seven patients treated with LT4 and monitored for thyroid carcinoma presented, in association with changes in LT4 dose, either with hypothyroid symptoms or symptom-free. Significant biochemical differences appeared between the different presentations. Leveled trajectories by subject to relief from hypothyroid symptoms differed significantly, indicating distinct responses, and denying a single shared outcome. These were formally defined by a high coefficient of the intraclass correlation (ICC1, exceeding 0.60 in all thyroid parameters) during follow-up on multiple visits at the same LT4 dose, when lacking symptoms. The intra-personal clusters were clearly differentiated from random variability by random group resampling. Symptomatic change in these patients was strongly associated with serum FT3, but not with FT4 or TSH concentrations. In 25 patients transitioning from asymptomatic to symptomatically hyperthyroid, FT3 concentrations remained within the reference limits, whilst at the same time marked biochemical differences were apparent between the presentations.

**Conclusions:** Considerable intra-individual clustering occurred in the biochemical and symptomatic responses to LT4 treatment, implying statistically multileveled response groups. Unmasking individual differences in the averaged treatment response hereby highlights clinically distinguishable subgroups within an indiscriminate patient panel. This, through well-designed larger clinical trials will better target the different therapeutic needs of individual patients.

## Introduction

Despite lacking the minor component of triiodothyronine (T3) physiologically co-secreted with thyroxine (T4) by the healthy human thyroid gland, in thyroid failure monotherapy by levothyroxine (LT4) replacement remains the standard treatment for patients with primary hypothyroidism (1, 2). LT4 is one of the most frequently prescribed drugs with a long history of successful use and favorable safety record (3–5). Administered in variable doses, dose adequacy for a hypothyroid patient is determined by biochemically defined treatment targets based mainly on TSH measurements (2). This marks a historical shift from earlier regimens primarily aiming at symptom relief (6). However, despite achieving appropriate biochemical treatment targets with LT4, as defined by current guidelines (2), a substantial fraction of patients continues to report persisting symptomatology expressing their dissatisfaction with the standard treatment (7). The magnitude of the problem has recently been re-emphasized by a large online survey conducted by the American Thyroid Association where satisfaction with LT4 treatment reported by the 12,146 respondents was only at median 5 on a scale of 1–10 (8). A prospective study by Winther et al. using a validated thyroid specific QoL questionnaire and following hypothyroid patients with autoimmune thyroiditis, concluded that QoL outcome measures improved but a full recovery was not achieved after 6 months of treatment with LT4 (9). While patients and doctors reported some success with the addition of T3 and guidelines by the European Thyroid Association acknowledge a potential benefit of T3/T4 combinations to some patients this subject overall remains contentious (7, 10–12).

Variable patient experiences with LT4 treatment and the possible existence of differently responding subgroups of patients have long been suspected (13), but formal analysis of this problem with robust statistical methods is seriously lacking. A biochemical dissociation in the equilibria or so called setpoint between TSH, FT4, and FT3 has been increasingly recognized, both in untreated subjects and in patients treated with LT4 (5, 13). Patients on LT4 display considerable variation in their biochemical and symptomatic treatment response, along with the manifestation of a pronounced disjoint between FT3 and TSH concentrations, compared to the relationship in thyroid health (14–16). This may also pertain to such intrinsic differences in patient response as to encourage an exploration of risk stratification. In this respect, the Rotterdam study documented an increased risk of both atrial fibrillation and sudden cardiac death in an untreated euthyroid population with higher LT4 serum concentrations within its reference range, yet uncorrelated with TSH concentrations (17, 18).

In the present study, we question to what extent the response to LT4 treatment, as expressed in the respective equilibria between the thyroid parameters TSH, FT4, and FT3, may differ between athyreotic patients with thyroid carcinoma and benign entities such as autoimmune thyroiditis or goiter post-surgery. In a panel of athyreotic patients with thyroid carcinoma followed long term on LT4 replacement, we assessed the biochemical alterations in individual subjects with symptoms before and after symptom relief. We were particularly interested in possible implications of ergodicity arising during long-term follow-up from a narrow intra-individual variation of thyroid hormones.

## Methods

### Patients

Data for this secondary analysis were collected as part of two previously reported trials, a cross-sectional prospective trial and a retrospective longitudinal study (19–21). The prospective trial was registered (www.ClinicalTrials.gov, NCT 01969552), ethically approved, and all participants gave written informed consent, and the retrospective study was approved by the local authorities in data protection. Both studies were conducted in an outpatient setting, prospectively from 2013 to 2014 in 1912 patients with various thyroid diseases and retrospectively from 2008 to 2016 in 319 patients with thyroid carcinoma routinely monitored at 2,309 visits (19, 21). Only LT4-treated out-patients without known comorbidity were included in the present study, and we also included only visits after hypothyroidism was biochemically controlled as defined by both a TSH < 4 mIU/l and FT4 > 10 pmol/l, while FT3 concentrations were within the reference limits. All measurements were obtained on unchanged stable medication in equilibrium. Indication for LT4 treatment resulted from three different indications, namely benign goiter, primary hypothyroidism due to thyroid autoimmune disease as evidenced by the presence of peroxidase antibodies (TPO Ab), and total thyroidectomy due to thyroid carcinoma. Patients with thyroid carcinoma were regularly monitored at 6 month intervals for the first 5 years after thyroidectomy and 12 month intervals thereafter in tumor-free patients, and followed long-term. Patient characteristics are tabulated as relevant for this study (Tables 1, 2).

**Table 1:** Patient characteristics in the cross-sectional study.

**Parameter**	**Goiter**	**Autoimmune thyroiditis**	**Thyroid carcinoma**	*P*-value^*^
Patients (*n*)	111	95	136	–
Gender (female/male)	83/17%	92/8%	71/29%	<0.001
Age (years)	59.5 (12.1)	51.9 (15.8)	54.6 (14.1)	<0.001
BMI (kg/m^2^)	26.8 [24.2, 30.8]	27.2 [23.5, 29.7]	28.3 [24.8, 33.0]	<0.001
Weight adjusted LT4 dose (μg/kg BW /day)	1.11 [0.88, 1.47]	1.27 [0.95, 1.67]	1.69 [1.52, 2.01]	<0.001
FT3 (pmol/l)	4.76 (0.52)	4.62 (0.56)	5.09 (0.72)	<0.001
FT4 (pmol/l)	17.15 [15.9, 19.6]	17.0 [15.1, 18.9]	20.4 [18.6, 22.8]	<0.001
TSH (mIU/l)	0.81 [0.42, 1.27]	1.32 [0.57, 2.04]	0.17 [0.03, 0.88]	<0.001

* *P-values were derived by ANOVA or, in case of non-normally distributed parameters, Kruskal-Wallis test*.

**Table 2:** Characteristics of patients with thyroid carcinoma in the longitudinal study.

**Parameter**	**Median [interquartile range]**
Patients (*n*)	319
Visits (*n*)	2,309
Follow-up duration (months)	63 [46, 81]
Follow-up intervals (months)	Six over the first 5 years, 12 thereafter, if tumor-free
Gender (female/male)	72/28%
Age at initial presentation (years)	50.1 [41.1, 62.0]
Body mass index (kg/m^2^)	28.2 [24.3, 31.3]
Tumor type	Papillary 69%, follicular 19%, other 12%
Tumor stage at initial presentation	pT1 46%, pT2 20%, pT3 12%, pT4 3%, N1 12%, M1 4%
Ablative treatment	surgery 100%, plus radioiodine 92.5%
Weight adjusted LT4 dose (μg/kg BW/day)	1.84 [1.62, 2.14]
TSH (mIU/l)	0.07 [0.01, 0.46]
FT3 (pmol/l)	5.15 [4.60, 5.80]
FT4 (pmol/l)	22.3 [19.6, 25.4]

Details were collected on patient history and medication, demographic factors (gender, age, BMI), physical examination, ultrasound, and laboratory tests (FT3, FT4, TSH, TPO Ab, and TSH-receptor antibodies (TSH R Ab) in TPO Ab positive cases only). In the longitudinal study only, any patient complaints, specific and non-specific, were freely communicated during visits in an open format, avoiding suggestive or standardized questions, and documented as such. The documented complaints were later independently categorized by a specialist into thyroid-unrelated symptoms (e.g., back pain), hypothyroid symptoms (e.g., tiredness, fatigue, lack of energy, cold intolerance, weight gain) and hyperthyroid symptoms (e.g., nervousness, irritability, restlessness, anxiety, rapid pulse, palpitations, trembling, heat intolerance, unwanted weight loss). The terms complaint and symptom are used synonymously here. During follow-up adjustments were made to the LT4 dose, either in response to individual patient complaints or according to the general treatment strategy including the individual risk profile and changes in guideline recommendations over the years (21).

### Thyroid Ultrasound

In all subjects, thyroid volume, echo-density, and nodularity were examined by ultrasound (10 MHz transducer). Thyroid volume was determined by the ellipsoid formula (longitudinal diameter × width × depth × 0.5 cm^3^) and summation of lobe volumes. Reference values are <18 ml for female and <25 ml for male subjects.

### Laboratory Methods

TSH was measured with an automated direct chemiluminescence method (TSH3-Ultra ADVIA Centaur XP, Siemens Healthcare Diagnostics, Erlangen, Germany). The standard curve was calibrated with the 3rd WHO Standard for hTSH (IRP 81/565). Functional sensitivity was 0.008 mIU/l, intra-assay variation 1.4–2.4%, and inter-assay imprecision 0.9–2.9%. FT3 and FT4 were measured on the same platform, showing intra-assay CVs from 2.4 to 3.1% or 2.2 to 3.3% and inter-assay CVs from 2.3 to 3.9% or 2.5 to 4.0%, respectively. Assay performance characteristics have been reported (22). Laboratory-evaluated reference intervals were as follows, 0.4–4 mIU/l for TSH, 3.1–6.8 pmol/l for FT3, 10–23 pmol/l for FT4.

TPO Abs were measured with a competitive chemiluminescence method (ADVIA Centaur XP, Siemens Healthcare Diagnostics, Erlangen, Germany) and TSH-R Abs with an ELISA (EUROIMMUN AG, Lübeck, Germany). Reference ranges were for TPO Ab <60 IU/ml and for TSH-R Ab <2 IU/l.

### Statistical Methods

Descriptive data are shown as mean (standard deviation, SD) or median (interquartile range, IQR). Non-normally distributed TSH values were natural logarithmically transformed. Between-two-group comparisons for continuous variables were based on Welch's *t*-test or, if normality could not be assumed, Wilcoxon's rank-sum test. More than three independent groups were compared using ANOVA or a Kruskal-Wallis test. Chi-squared test with Yates' correction for continuity was used for categorical variables. Pooled observations at either symptomatic or asymptomatic presentations derived from the same patient were compared using a paired *t*-test or the signed rank Wilcoxon test. Multivariable and multivariate linear models, adjusted for disease entity, gender, age, and BMI were used to assess associations across subjects between thyroid parameters including–when significant–more complex multiplicative interactions between them. MANOVA tests for the multivariate models relied on Pillai's test statistic. Residual plots were inspected to verify model assumptions. Changes during follow-up in the binary outcomes for the presence or absence of symptoms and continuous thyroid parameters were assessed using generalized linear mixed models with a restricted maximum likelihood estimator (REML) and a binomial or Gaussian link function, respectively, appropriately accounting for within-variation and intra-subject correlations for repeated measurements per subject in the longitudinal design (23). Effect plots predict the binary outcome as a probability response on a linearized logit scale or the natural response of a continuous outcome. Relative risks (RR) are reported in Results. Model performance was compared by both *F*-test and Akkaike's information criteria (AIC). These models were formulated as unconditional means models to derive estimates on intra-class correlation coefficients (ICC1) and reliability (ICC2) for thyroid hormones obtained at multiple occasions under stable conditions during follow-up. Random group resampling was performed to differentiate personal group-level properties from random group variability (24, 25). Power simulations were done according to the methods described and implemented by Bliese (24, 25). All tests were two-sided with *p* < 0.05 denoting statistical significance. Variables were considered explanatory without adjusting for multiple comparisons. We used the R statistical software environment (version 3.5.2 for Mac) (26) with the added packages lme4 1.1-19 (23), effects 4.1-0 (27), heplots 1.3.-5 (28), sjstats 0.17-3 (29), and multilevel 2.6 (24, 25).

## Results

Patient characteristics of LT4-treated patients are summarized in Table 1 for the cross-sectional study and in Table 2 for the longitudinal study.

### Cross-Sectional Study

**Figure 1:**
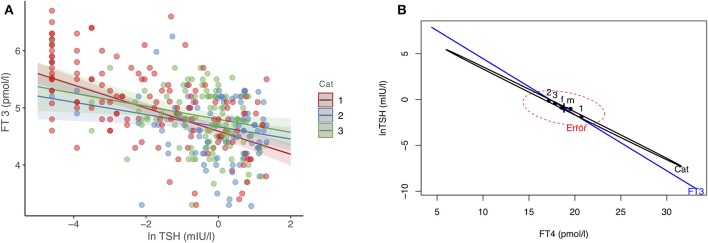
**(A,B)** Effect plots of the complex interdependency between FT3, FT4, and TSH. **(A)** shows the relationship between FT3 and TSH, examined by treatment category, after adjusting for FT4 concentrations, gender, age, and BMI. **(B)** The resulting equilibria between FT4 and TSH (as surrogate markers for setpoints), associated with treatment category and FT3 concentrations in a significant interaction (Pillai-test *p* < 0.001, see Results for details). The three treatment categories refer to patients with thyroid carcinoma (1), autoimmune thyroiditis (2), and benign goiter (3). f indicates female, and m male gender. Cat, disease category. Regression lines are surrounded by the 95% confidence limit.

In 342 patients treated with LT4, we examined their equilibria and biochemical response heterogeneity by disease entity, benign goiter (*n* = 111), autoimmune thyroiditis (*n* = 95), thyroid carcinoma after thyroidectomy (*n* = 136). Using FT3 levels achieved as dependent outcome in a multivariable linear model, disease category (significantly steeper relationship in the carcinoma group, *p* = 0.02), FT4 (0.03 pmol/l per pmol/l, 95% CI [0.014, 0.052], *p* < 0.001), and TSH (lnTSH −0.17 pmol/l per mIU/l, 95% CI [−0.21, −0.13], *p* < 0.001) concentrations were all significantly independent predictors. The influence of the other adjusted covariates present in the model was as follows, gender (0.42 pmol/l higher for men, 95% CI [0.29, 0.56], *p* < 0.001), age (−0.012 pmol/l per year 95% CI [−0.015, −0.008], *p* < 0.001), BMI (−0.002 pmol/l per kg/m^2^, 95% CI [−0.011, 0.006], *p* = 0.60). Figure 1A shows the TSH-dependent and FT4-adjusted FT3 response by treatment category. Conversely, in a multivariate model, FT3 concentrations interacted with the treatment category in predicting the combined outcomes for FT4 and TSH, used as surrogates for setpoints. This interaction was highly significant (Pillai test, *p* < 0.001), and remained so after adjusting for gender, age and BMI (Pillai test, *p* < 0.001). Figure 1B shows the derived trajectories for the relationships and estimates the resulting FT3-dependent equilibria (setpoints) between FT4 and TSH serum concentrations by treatment category and FT3 levels.

### Longitudinal Study

**Figure 2:**
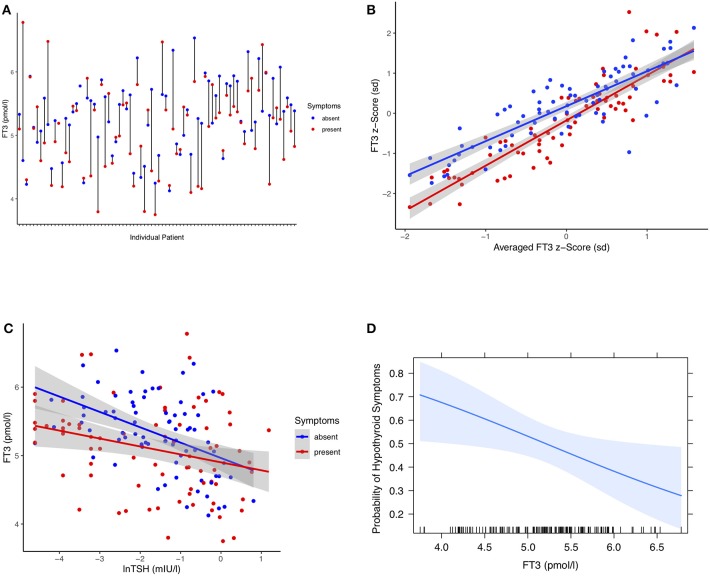
**(A)** Difference plot of serum FT3 concentrations in 77 individual patients at either visits with hypothyroid symptoms or asymptomatic presentations. Each point refers to pooled measurements obtained from a single patient during follow-up and averaged over multiple either symptomatic or asymptomatic visits after adjusting LT4 dose (see Results). **(B)** FT3 z-scores of either symptomatic or asymptomatic presentations plotted against the averaged scores over all visits per patient. FT3 concentrations are mean-centered and units are expressed in standard deviations. This shows that the corrective FT3 difference associated with relief of self-reported hypothyroid symptoms increased progressively with increasing distance from the center. Shaded areas indicate the 95% confidence limit for the fitted regression line. **(C)** FT3 change relative to TSH stratified by symptomatic and asymptomatic presentations of the same patients. The intersecting points did not move along a shared trajectory between the two conditions but were significantly shifted, progressively so toward lower TSH concentrations (see Results). **(D)** Probability of hypothyroid symptoms as a function of circulating FT3 concentrations. The probability of the presence of hypothyroid symptoms at a given FT3 level for these patients was derived by a multilevel model accounting for intra-class and between-subject variation (see Methods and Results). The shaded area indicates the 95% confidence limit of the probability curve. The vertical ticks on the x axis indicate the observed individual values.

In a longitudinal series of 319 patients with thyroid carcinoma followed at 2,309 visits for 63 months (median, IQR 46, 81), we assessed the individual treatment responses. Of particular interest were 77 patients with changing hypothyroid symptomatology following LT4 dose adjustment during follow-up. The statistical comparison between pooled paired observations, averaged over multiple visits (median 9, IQR 6, 12) at either symptomatically hypothyroid or asymptomatic presentations, was as follows, weight-adjusted LT4 dose (−0.081 μg/kg BW, 95% CI [−0.14, −0.024], paired *t*-test: *p* = 0.006), TSH concentrations (0.14 mIU/l [−0.03, 0.31], paired signed Wilcoxon test: *p* = 0.47), FT4 concentrations (−1.05 pmol/l, 95% CI [−1.83, −0.28], paired *t*-test: *p* = 0.009), and FT3 levels (−0.22 pmol/l, 95% CI [−0.36, −0.08], paired *t*-test: *p* = 0.002), all except TSH being significantly lower at presentations with hypothyroid symptoms. A difference plot of FT3 measurements between the symptomatic and asymptomatic pairs revealed considerable diversity among individual patients in their start and end levels and respective distances between the two levels (Figure 2A). After mean-standardizing the FT3 concentrations, we plotted the z-scores for FT3 of either symptomatic or asymptomatic presentations against the average scores over all visits per patient (Figure 2B). This shows that the corrective effect size required for relief of hypothyroid symptoms increased with the distance from the center. Looking at FT3 change relative to TSH, symptomatic and asymptomatic observations did not move along a shared trajectory but were significantly shifted (0.23 pmol/l 95% CI [0.36, 0.11], *p* < 0.001) (Figure 2C). On average, the rate of hypothyroid symptoms increased with lower FT3 concentrations (Figure 2D). FT3 serum concentrations (RR 0.70 per pmol/l, 95% CI [0.49, 0.96], *p* = 0.03), but not FT4 concentrations (RR 0.95 per pmol/l, 95% CI [0.91, 1.00], *p* = 0.053), and TSH concentrations (lnTSH RR 0.98 per mIU/l, 95% CI [0.87, 1.09], *p* = 0.73) were significantly predictive of the presence of hypothyroid symptoms in these patients. In this respect, a combination of all three covariates FT3, FT4, and TSH was not more informative, compared to FT3 measurements alone (*F*-test *p* = 0.14, AIC difference 0.11). Confounders in the cross-sectional study, namely gender (*p* = 0.30), age (*p* = 0.57), and BMI (*p* = 0.93), were non-influential but adjusting for these covariates slightly reduced the variation of the significant FT3 influence (adjusted RR 0.55, 95% CI [0.31, 0.87], *p* = 0.008).

**Figure 3:**
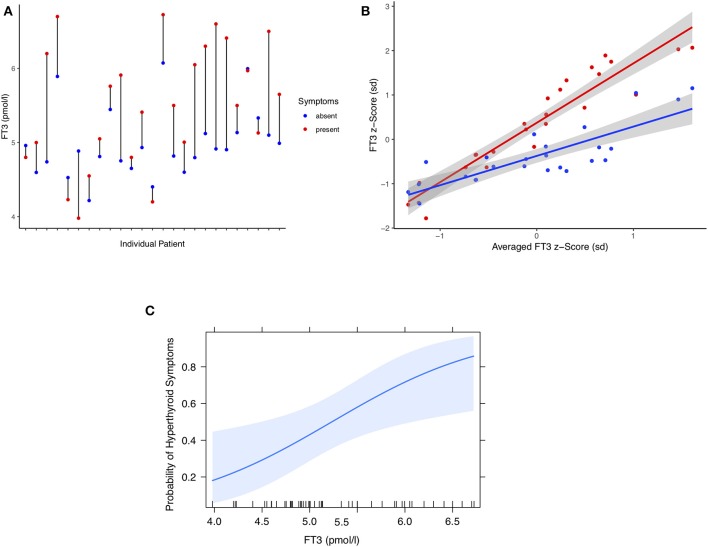
**(A)** Difference plot of serum FT3 concentrations in 25 individual patients at either hyperthyroid or symptom-free presentations. Each point refers to the pooled measurements over multiple either symptomatic or asymptomatic visits (see Results). **(B)** FT3 z-scores of either symptomatic or asymptomatic presentations plotted against the averaged scores over all visits per patient. Shown are mean-centered standardized FT3 concentrations and a fitted regression line surrounded by its 95% confidence limit (shaded area). **(C)** Probability of hyperthyroid symptoms in these patients as a function of circulating FT3 concentrations. A multilevel model was used to estimate the probability (see Methods and Results). The shaded area indicates the 95% confidence limit of the fitted curve. The vertical ticks on the x axis indicate the observed individual values.

Similar to the hypothyroid complaints, the pooled observations from 25 individual patients were compared when they were either symptom-free or presented with hyperthyroid symptoms (Figures 3A–C). Differences were highly significant for weight-adjusted LT4 dose (0.23 μg/kg BW, 95% CI [0.12, 0.35], paired *t*-test: *p* < 0.001), TSH concentrations (−0.44 mIU/l [−0.60, −0.29], paired signed Wilcoxon test: *p* < 0.001), FT4 concentrations (4.20 pmol/l, 95% CI [2.83, 5.58], paired *t*-test: *p* < 0.001), and FT3 levels (0.53 pmol/l, 95% CI [0.26, 0.80], paired *t*-test: *p* < 0.001). For individuals, FT3 and symptomatic change are shown in Figure 3A. Standardized effect sizes are depicted in Figure 3B. The probability of hyperthyroid symptoms increased with higher serum concentrations of FT3, as shown in Figure 3C. Relative risk estimates with increasing concentrations were as follows, FT3 1.54 per pmol, 95% CI [1.13, 1.79], *p* < 0.001), FT4 1.17 per pmol, 95% CI [1.06, 1.28], *p* = 0.003), and lnTSH 0.42 per mIU/l, 95% CI [0.24, 0.70], *p* < 0.001).

### Intra-Class Correlations and Reliability

We estimated the intra-class correlation coefficient (ICC1) and reliability (ICC2) over the follow-up period in 141 patients at 435 visits on a stable unchanged LT4 dose of 125 μg/day and in the absence of symptoms. The individual patients displayed a high intraclass correlation for all thyroid parameters, FT3 0.61, FT4 0.67, and TSH 0.67. FT3-dependent multilevel trajectories to relief of hypothyroid symptoms also proved highly individually variable, ICC1 0.64.

All parameters showed excellent group-mean reliability FT3 0.83, FT4 0.86, TSH 0.86, indicating that the individuals do not form random groups and can be reliably differentiated. To estimate how much appropriately accounting for level properties may reduce variance or, conversely, if ignored, inflate variance we compared the variance of randomly resampled pseudo-groups with the real variance in the actual groups where every patient formed their own group during follow-up. This demonstrates a highly significant (*p* < 0.001) and pronounced influence of personal grouping, as opposed to random grouping, the mean within-group variance for the random FT3 sample being 0.46, compared to 0.26 for the real data for FT3.

### Potential Bias of Ignoring Intra-Class Correlations

A bias may arise, for instance in an RCT, if the individual levels are disregarded and the data is treated as though it was independent. Simulating a hypothetical design with two groups, 40 subjects per group, moderate between-variable correlation (*r* = 0.47) and intra-class correlation of 0.61 for the outcome variable, the *t*-test-based power estimate of such a trial would be reduced from 92 to 48% if the analysis fails to account for the observed multilevel structure in the data. A sufficiently powered (93%) larger trial with a group size of 200 subjects and a lower correlation of 0.25 under otherwise identical conditions would become underpowered by ignoring the level properties in the sample (power estimate 63%). Increasing group size to 500 subjects in the case of three groups and two weakly correlated variables (*r* = 0.10) would not remedy the lack of power caused by averaging (54%), compared to leveling (91%) the outcome.

## Discussion

Although in health a narrow intra-individual variation of thyroid hormones has long been recognized (30), its application to the treatment of patients with LT4 has not been rigorously examined. This study has uncovered considerable inter-individual variability and intra-class correlations in the biochemical and symptomatic responses to LT4 treatment within a patient panel. For instance, failure to account for a multilevel structure in the data, if present in a randomized controlled clinical trial (RCT), may mask potential treatment effects. This will result in the reduction of statistical power when predicting treatment-associated outcomes in hypothyroid patients on LT4.

### Non-ergodicity of Thyroid Parameters

In probability theory, the definition of an ergodic dynamical system is that it displays the same behavior when averaged over time as averaged over the space of all the system's states in its phase space (31). The mathematical definition emphasizes that for group membership all group members must share the same moments, namely means, variances and covariances (32). Ergodicity is a requirement when generalizing from the population to the individual level (32–35). Because the implicitly assumed ergodicity of thyroid parameters does not hold true, thyroid reference ranges are fundamentally inappropriate and should be replaced by personal setpoints (5). Ergodicity is most particularly challenged in situations where both trait-like differences exist among individuals and structural change occurs over time. In thyroid patients, the concept relates to trait-like personal setpoints (equilibria between TSH and FT4, FT3) that undergo structural change during follow-up. The determination of intra-class correlations (ICC) provides a quantitative measure on influences associated with either a subject or a particular situation.

### Intra-Class Correlation of Thyroid Parameters

In our longitudinal series, we documented substantial intra-class correlation for all thyroid parameters. This characterizes the thyroid status largely as a personal trait, which varies under stable medication more between subjects, and less so within a person on different occasions. Random variability was ruled out by a permutation test, confirming both the multi-level properties and individual heterogeneity among the patients within the panel. Whenever the intra-class correlation coefficient is found to be large, we cannot confidently use aggregated statistical methods on these data that assume independence, because estimates of variance, and therefore *p*-values, become insufficiently robust. As pointed out by Fisher et al., best-practice guidelines derived from RCTs in such conditions tend to overestimate the accuracy of aggregated statistical estimates (35). Ecological fallacy, collider stratification bias and Simpson's paradox are variations of the problem with serious implications, as exemplified by the market retraction of an approved drug (34–38).

Setpoint theory may explain the high degree of individuality physiologically observed in thyroid parameters (39, 40). In the thyroid healthy state, the so-called setpoint delivers a homeostatically defined multivariate expression of the stable equilibrium between interlocked pairs of TSH and FT4 (41). The resulting distribution of clustered setpoints is fundamentally different from the process of using univariate reference ranges for TSH or FT4, rather requiring multivariate and multileveled approaches (5, 41). In the event of a disease or under the influence of LT4 treatment, personal set points may be conditionally redistributed (13, 14). Consequently, the TSH level previously appropriate for thyroid health cannot equally serve as a treatment target in the same person (42).

From an evolutionary point of view, moderate diversity in their personal setpoints and response heterogeneity among individuals within a human or animal population makes sense, attenuating excessive reactions, and abrupt transitions to changing environmental conditions in a community (43–45).

### Intra-Class Correlation and Patient Complaints

The reasons for persisting patient complaints are not well-understood and thyroid-related symptoms may overlap with a plethora of non-specific complaints (8, 46–60). Also, drugs containing LT4 or LT3 may display mild antidepressant pharmacological properties (61). In an ergodic framework, we may question whether complaints relate to the dynamic structure of the underlying thyroid process or are independent “traits” of the individual and derive quantitative estimates for the two components. Concerns about inexplicable variation (62, 63) should be advanced from the descriptive level to analytical study. A focus on idiographic patterns following individual patients receiving LT4 long term on multiple occasions, as in this retrospective study, limits the impact of inter-personal variation. It reduces thereby the importance of chief non-thyroidal confounders of cross-sectional studies such as gender, age and BMI as well as treatment-related variation in the biochemical equilibria across different disease entities, such as thyroid carcinoma, autoimmune thyroiditis, and goiter. This may uncover subtle differences that may otherwise remain hidden within a noisy background and go undetected by statistically inappropriate averaging.

### Strengths and Limitations of the Study

The present study is one of the first of its kind in conducting an intra-class correlation analysis in the treatment response to LT4 over multiple presentations in a large sample of patients with thyroid carcinoma followed for several years. It has however several limitations. This is a secondary analysis; the primary clinical study outcomes of both the prospective cross-sectional trial and retrospective longitudinal study have previously been reported (19, 21). Although patients with known comorbidities, interfering comedication, and clinical conditions in which elevated TSH levels persisted (e.g., non-adherence, LT4 malabsorption) were excluded from this analysis remaining subclinical pathologies are part of the biological variation (64). The present analysis focusses on the framework of ergodicity and multileveled patterns of the responses to LT4 treatment within a patient panel. Although the study design was uncontrolled, the findings of the ICC analysis are pertinent to prospective studies and RCTs and may aid in improving future trials. As hypothyroid symptoms inherently overlap with non-specific or hyperthyroid complaints, a few misclassifications are inevitable but are of little apparent influence on the main tendencies for each symptom category (Figures 2, 3). More importantly, patient expectancy remains a bias that has not been robustly addressed in any thyroid trial including RCTs (65). The American Food and Drug Administration (FDA) therefore demands drugs to be evaluated under “actual conditions of use”–a requirement met by none of the many RCTs on QoL outcomes for LT4 and T4 T3 combinations (10, 11, 65).

### Symptom Evaluation

Subjective symptoms as experienced by the patient were freely communicated during routine visits in an open format, being retrospectively and independently categorized into hypothyroid, hyperthyroid, or thyroid-unrelated complaints. While unstandardized, this process avoids any suggestive interrogation. The presence of leading symptoms may successfully substitute for the use of more complex QoL questionnaires, and instantaneous assessment offers higher precision and sensitivity to change, compared to retrospective ratings (66, 67). Due to their non-ergodic behavior TSH and thyroid hormone levels associated with the presence or absence of hypothyroid symptoms considerably overlap among individual patients. Individual trajectories to symptom relief start from different levels and end at different targets. Intra-class clustering and shifts in the treatment response reduce the discriminatory power of averaged between-subject comparisons in trials, including RCTs, as demonstrated in Results.

T4 mainly acts as a circulating pro-hormone, requiring both prior transmembrane transport and enzymatic activation to exert a multitude of genomic actions through nuclear thyroid hormone receptor binding (68–71). Both T3 conversion rates and thyroidal T3 secretion are subject to central control by TSH (72–76). The loss of functioning thyroid tissue and/or the TSH-lowering effect of LT4 treatment may impair this compensatory mechanism (16, 19, 77). Due to the expression of distributional individuality and subsequent disruption of the TSH-FT4 correlation, subclinical hypothyroidism is an indeterminate and unreliable disease classifier (5, 13). This dissolves the existing relationships in an individual prior to thyroidectomy, re-adjusting the setpoint and re-setting the equilibria between TSH, FT4, and FT3 in thyroid disease, compared to the healthy state (14, 15, 42, 78). While regarded as essential in maintaining narrow individual serum concentrations of the respective hormones (30), the non-ergodic behavior has yet to be transferred to the treatment situation where population range-based recommendations are paramount in guidelines (2).

### Clinical Implications

Dissimilar clusters or individuals may have conditional requirements for optimum treatment success different from the averaged population and risk profiles may also be shifted. This is in accord with a recent prospective study defining the optimum TSH target slightly below the lower reference limit for patients with thyroid carcinoma treated with LT4, based on the examination of surrogate markers (79). TSH-independent risk profiles have also been demonstrated by the Rotterdam study in euthyroid subjects, although this study did not include a sufficient number of LT4-treated subjects (17, 18). We note that in our study patients with uncontrolled or refractory hypothyroidism (TSH > 4 mIU/l) were excluded and TSH suppression was primarily motivated by tumor control–which is now managed differently–not symptom control (80–83). We do not infer that patients should have a suppressed TSH, rather that the personal levels expressed in the treated condition have different meanings, compared to the untreated situation. Neither does a TSH measurement within its reference limits guarantee that a patient will be symptom-free, nor that a presumably healthy person by this definition may not suffer serious adverse consequences such as atrial fibrillation (17). We and others proposed a more personal definition of “euthyroid,” based on individual traits (setpoints) and dynamic changes between the relationships of all three thyroid parameters TSH, FT4, and FT3 (5). This extends to both genetically determined fingerprints and treatment-related alterations in the expression of personal setpoints and includes other allostatic expressions of individuality (e.g., in their gut microbiome) affecting iodothyronine homeostasis (84–90).

Dissimilarities in the treatment responses between individual patients are particularly apparent when patients on LT4 display substantial variation in their T4–T3 conversion efficiency and pronounced disjoints between their serum TSH and FT3 concentrations (16, 77). Importantly, FT3 levels relative to TSH in symptomatic vs. asymptomatic presentations of the same patients did not move along a shared trajectory but were shifted upward when the patients transitioned from the symptomatically hypothyroid to the asymptomatic condition. Depending on the patient presentation, the addition of LT3 is increasingly considered by thyroid experts worldwide (12). Differential treatment of identifiable dissimilar subpopulations, e.g., with persistently low FT3 concentrations despite normalized TSH (77), appears feasible, but was not tested in this study and awaits further proof.

## Summary

Complex patterns emerge between TSH, FT4, and FT3 in patients treated with LT4 during follow-up in response to the treatment and changes in LT4 dose, displaying a high degree of intra-class correlation and multileveled structure. This invokes the danger of inappropriate statistical averaging in clinical trials, mandating a stronger focus on within-subject analyses according to ergodic principles. It emphasizes a need to better define personal treatment outcomes and individual risk profiles in patients receiving LT4 alone or, similarly, a combination of T3 and T4.

## Ethics Statement

The prospective trial was registered (www.ClinicalTrials.gov, NCT 01969552) and the protocol was ethically approved by the Ethical Committee of the University of Münster, Germany. All participants gave written informed consent. The retrospective analysis was specifically approved by the local authorities in data protection. The study was carried out in accordance with the Declaration of Helsinki.

## Author Contributions

All authors have significantly contributed to the findings reported here, and all authors have jointly conceptualized the study and agreed to the final submitted manuscript.

### Conflict of Interest

JD is co-owner of the intellectual property rights for the patent “System and Method for Deriving Parameters for Homeostatic Feedback Control of an Individual” (Singapore Institute for Clinical Sciences, Biomedical Sciences Institutes, Application Number 201208940-5, WIPO number WO/2014/088516). The remaining authors declare that the research was conducted in the absence of any commercial or financial relationships that could be construed as a potential conflict of interest.
